# Progress in Gynæcology

**Published:** 1904-02-06

**Authors:** 


### PROGRESS IN GYNÆCOLOGY.

**Tuberculosis of the Uterus and Adnexa.**—During the last few years considerable advance has been made in our knowledge of tubercular disease of the uterus and its adnexa. J. H. Targett[1] considers the following to be the principal modes of infection:—1. By the blood stream, the original infection being in the lymphatic glands, particularly the bronchial. 2. By the peritoneum in cases of general miliary tuberculosis. 3. By direct infection from neighbouring organs—*i.e.*, the ileum, the rectum, and the bladder. Tuberculosis of the body of the uterus may be diffuse miliary and non-ulcerative, or granular, friable, and caseous; or there may be pyometra from the cervical canal being blocked. In the majority of cases of tuberculosis of the uterus the infection comes from the tubes; these being affected in 90 per cent. of all cases involving the genital organs. The salpingitis may be miliary or caseous, and the tube when pus accumulates in it assumes a peculiar banana-like shape. Amenorrhœa is not uncommon, but when the periods are present dysmenorrhœa is the rule. With regard to treatment, laparotomy is often beneficial, but removal of the tuberculous organs is generally very difficult on account of adhesions, and it is sometimes followed by rapid toxæmia. A. W. Lea[2] reports five cases of tubercular disease of the tube treated by operation, and says the diagnosis of removed specimens should never be made without a microscopic examination, as nodules having a naked eye resemblance to those of tubercle are sometimes met with in other conditions. It is never safe to rely on the presence of bacilli in the vaginal discharge. Spontaneous cure by the development of fibrous tissue is in some cases possible.

**Rupture of Uterus following Caesarian Section.**—A very interesting case of rupture of the uterus following Caesarian section is reported by Füth.[3] At the age of 24 the patient had Caesarian section performed on account of a dermoid ovarian cyst which was impacted in the pelvis and obstructed the passage of the child. Two years after this she was admitted into the hospital with complete rupture of the uterus, apparently at full term. The abdomen was opened, when the fœtus and placenta, with the membranes entire and unruptured, were found in the peritoneal cavity amongst the coils of intestines. The uterus was empty and had a crater-like opening on its anterior surface as large as the palm of the hand. The rupture had not occurred at the site of the old incision in the uterus, the scar of which was distinctly recognisable. There was no evidence that a tubo-uterine pregnancy had caused the rupture, and it was thought to be probably due to the first operation having somewhat weakened the uterine muscle or altered its action. Supra-vaginal amputation was performed, with drainage through the cervical canal. The patient remained in a very critical state for three or four days, but ultimately made a good recovery.

**Diagnosis and Treatment of Uterine Fibroids.**—Murdock Cameron[4] in an instructive paper on this subject says that he does not consider that these tumours are more common in single than in married women, and he looks upon sterility as a consequence rather than a cause. Some fibroids are so soft in consistence that they may easily be mistaken for ovarian cysts. Most fibroids increase in size during pregnancy, and subsequently diminish during the involution of the uterus. They undergo a certain amount of atrophy at the menopause, but rarely disappear altogether. Subserous tumours, except from torsion of the pedicle give rise to few symptoms. Submucous and interstitial ones cause bleeding, generally menorrhagia, but sometimes metrorrhagia. When no urgent symptoms are present he advises that interference should be delayed. When operation becomes necessary, hysterectomy is the most satisfactory proceeding, enucleation and oöphorectomy being now almost abandoned.

**Posterior Displacements of the Uterus.**—There is still considerable difference of opinion as to the relative value of vaginal and abdominal section in the treatment of retrodisplacement of the uterus complicated by adhesions, some operators strongly advocating the vaginal operation, while others are equally in favour of the abdominal. E. H. Grandin[5] is strongly in favour of the latter route; he says that in these cases the uterus, tubes, and ovaries, are often matted together, and sometimes adherent to the vermiform appendix and intestine, and that to expose the patient to the risk of dealing with these complications through a comparatively small vaginal opening is quite unjustifiable. J. Riddle Golfe,[6] on the other hand, considers that through a vaginal incision any adhesions can be safely dealt with, and the uterus restored to its normal position. He points out that the uterus is maintained in its normal position by means of ligaments (of which the utero-sacral, the utero-vesical, and the round ligaments are the most important) and it is to operations that will restore these structures to their normal condition, that we must look to cure retrodisplacements of the uterus, and all these structures, he maintains, can be reached through a vaginal incision.

[1] Brit. Med. Jour., Aug. 8, 1903. [2] Lancet, Aug. 15, 1903. [3] Quarterly Med. Jour., May, 1903. [4] Brit. Med. Jour., Aug. 8, 1903. [5] Med. Rec., March 14, 1903. [6] Ibid.

*(To be continued.)*

